# Quality of care before and after initial certification at a German certified hereditary breast and ovarian cancer center

**DOI:** 10.1007/s00432-025-06388-3

**Published:** 2025-12-17

**Authors:** Niklas Amann, Manuel Hörner, Henrik Spannring, Lena Brückner, Julia Gocke, Carolin Müller, Cornelia Boesl, Simon Bader, Felix Heindl, Carolin C. Hack, Peter A. Fasching, Matthias W. Beckmann, Annika Krückel

**Affiliations:** 1 Department of Gynecology and Obstetrics, Universitätsklinikum Erlangen, Comprehensive Cancer Center Erlangen-EMN (CCC ER-EMN), Universitätsstraße 21/23, 91054 Erlangen, Germany; 2 Friedrich-Alexander-Universität Erlangen-Nürnberg (FAU), Erlangen, Germany; 3 Bavarian Cancer Research Center (BZKF), Erlangen, Germany; 4 Department of Gynecology and Obstetrics and CCC Munich LMU, LMU University Hospital, Munich, Germany

**Keywords:** BRCA, Breast cancer, Certification, Genetic testing, HBOC, Ovarian cancer

## Abstract

**Purpose:**

Genetic mutations contribute to around 10% of breast and 25% of ovarian cancers, with one third of patients having a familial cancer history. The German Consortium for Familial Breast and Ovarian Cancer was founded in 1996 to improve care for these patients. Certification of cancer centers, introduced in 2004, has been linked to improved survival rates and ensures adherence to evidence-based standards. This study investigates changes in care structures and quality before and after the initial certification of the HBOC center at the University Hospital Erlangen, certified from 2021 on.

**Methods:**

This retrospective study analyzed patient data from January 2018 to December 2023 at the certified Hereditary Breast and Ovarian Cancer center at the University Hospital Erlangen. Eligibility for genetic counseling and germline testing followed the German Cancer Society criteria. After informed consent, Next Generation Sequencing was performed, and variants were classified according to Human Genome Variation Society and American College of Medical Genetics and Genomics standards. Medical histories and genetic results were recorded in electronic case report forms.

**Results:**

From 2018 to 2023, a total of 2694 genetic tests were performed, increasing from 962 pre-certification to 1732 post-certification (+ 180%). Testing among affected female patients doubled. Genetic testing in breast cancer patients increased from 551 to 1,04, while testing for ovarian carcinoma rose from 117 to 159. Variants of uncertain significance were identified in approximately 9% of cases during both periods. Pathogenic findings were observed in 14.3% of cases pre-certification (with 9.2% involving *BRCA1/2* mutations) and 11.5% post-certification (6.4% *BRCA1/2* mutations). Enrollment in the intensified surveillance program (IBCS) increased by 182.5%, accompanied by a rise in recommendations for risk-reducing surgeries.

**Conclusion:**

Certification of medical institutions ensures high-quality, evidence-based patient care and increases the utilization of preventive and counseling services, particularly for Hereditary Breast and Ovarian Cancer. Further studies are needed to confirm the long-term impact and necessity of certification.

**Supplementary Information:**

The online version contains supplementary material available at 10.1007/s00432-025-06388-3.

## Introduction

Breast cancer is the most common oncological disease among women worldwide (Harbeck et al. 2019). In Germany, more than 70,000 women are diagnosed with this disease each year (Tauber et al. 2025). In approximately 10% of patients, the cancer is attributable to a genetic mutation in genes associated with hereditary breast and ovarian cancer (HBOC) (Beckmann, et al. 1997; Ataseven et al. 2020). The development of ovarian cancer is similarly influenced by genetic alterations, which can be detected in over 25% of cases (Harter et al. 2017). Consequently, one third of all patients with breast or ovarian cancer have a familial history of cancer (Kast et al. 2016).

To identify these patients and provide appropriate care, the German Consortium for Hereditary Breast and Ovarian Cancer (GCHBOC) was established in 1996 (Klein et al. 2024). Over the past three decades, comprehensive structures have been developed to offer patients optimal counselling. The clinical implications of a familial high-risk situation, either due to the detection of a pathogenic germline mutation or the presence of a significant family history indicating an elevated risk despite unremarkable germline testing, may include not only enrollment in an intensified surveillance and follow-up program (IBCS), but also recommendations for risk-reducing surgical interventions (e.g., risk-reducing bilateral mastectomy (RRBM), risk-reducing salpingo-oophorectomy (RRSO)). Genetic testing (predictive and diagnostic) is also performed in human genetics outpatient departments. However, the centers of the GCHBOC have established contracts with German health insurance providers, stipulating that counseling and testing of patients with HBOC, as well as their subsequent management within the framework of IBCS, can only be reimbursed when conducted at university hospitals or GCHBOC-affiliated centers.

To ensure the quality of oncological care, breast cancer centers in Germany were first certified by a national commission in 2004 (Beckmann et al. 2011). The WiZen study demonstrated that cancer patients treated in certified cancer centers exhibit improved overall survival rates (Schmitt et al. 2023). These findings underscore the importance of adhering to evidence-based standards in the treatment of oncological patients. However, the WIZEN study did not include unaffected individuals with an increased risk of cancer. Patients with HBOC were not examined in this analysis.

As part of the certification process, specific targets and requirements are established to ensure the necessary level of expertise (Wesselmann et al. 2014a; Kowalski et al. 2017). In addition to core data, such as genetic counseling and testing of both affected and unaffected individuals, metrics including the number of newly diagnosed breast cancer cases within the framework of the early detection program, the proportion of pathogenic mutations, and the number of initial studies conducted are systematically recorded (DKG 2025) (Supplementary Table 1.). Annual follow-up re-certifications are intended to ensure the high standards established during the initial certification and to contribute to the continuous improvement of care structures and quality within the centers (Wesselmann et al. 2014a).

In 2004, a center specializing in Hereditary Breast and Ovarian Cancer (HBOC) was established for the first time at the Department of Gynecology and Obstetrics, University Hospital Erlangen. This center was integrated into the GCHBOC in 2019. From 2021 ongoing, the HBOC center at the University Hospital Erlangen was certified for the first time by the German Cancer Society. Since then, annual re-audits have consistently ensured the maintenance of high standards of care quality, in line with the certification requirements for centers specializing in HBOC.

The primary objective of the study is to compare the absolute number of patients before and after certification. Secondary objectives include analyzing patient characteristics, tumor types, and germline testing results, with a focus on their preventive and therapeutic implications. Furthermore the study aim, to determine the extent to which structural, process, and outcome quality could be influenced and to assess how care structures evolve at a certified center in the years following initial certification.

## Methods

### Patient cohort

Between January 1, 2018, and December 31, 2023, 2694 patients presented at the HBOC center at the University Hospital Erlangen. In order to qualify for genetic counseling related to HBOC, patients must fulfill the testing criteria established by the German Cancer Society. A distinction is made between affected and unaffected individuals, whereby there may be preventive, diagnostic, or therapeutic indications for germline testing. Patients diagnosed with breast or ovarian cancer must also meet the German Cancer Society’s testing criteria. Genetic testing is recommended if breast cancer is diagnosed before the age of 36, triple-negative breast cancer age cut off changed over the past or ovarian cancer before the age of 80, even in the absence of a suspicious family history. These criteria are reviewed annually and adapted based on the latest scientific evidence, resulting in changes to the testing guidelines during the study period. For the treatment of metastatic or locally advanced HER2-negative breast cancer, germline testing also gained therapeutic indication.

To collect data on first diagnoses of ovarian at the Gynecologic Cancer Center and breast cancer at Breast Cancer Center of the University Hospital Erlangen at all, figures from the publicly available OnkoZert certification reports for the years 2018 to 2023 were used.

### Structured processes

Patients eligible for genetic counseling are identified through a systematic assessment of the German Cancer Society’s inclusion criteria. If the criteria are met, patients can undergo counseling at the HBOC center. The counseling is provided by trained medical personnel who have completed the GCHBOC basic module and hold additional qualifications in specialized human genetics counseling.

**Fig. 1:**
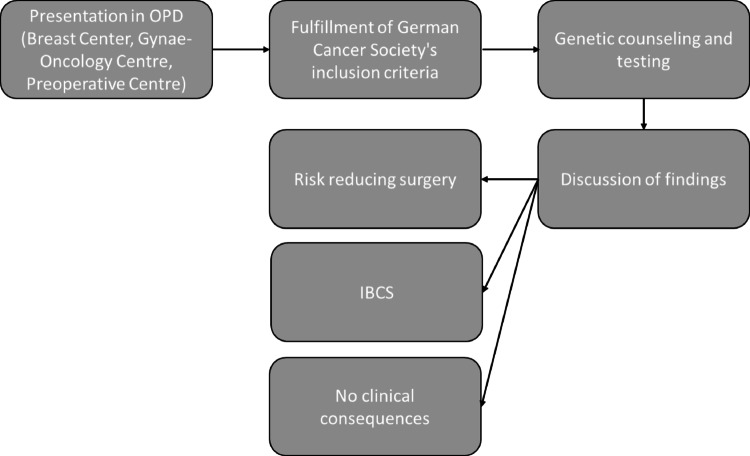
Structured process of genetic testing at the Hereditary Breast and Ovarian Cancer Center at the University Hospital Erlangen

Upon completion of the counseling session and after obtaining written informed consent, genetic testing is initiated (Fig. 1.). A blood sample is collected and analyzed using Next Generation Sequencing (NGS) technology. The specific methods employed are detailed in the molecular genetic report, which also includes information about the genes analyzed and any identified genetic alterations. More than one genetic variant could be possible. Genetic variants are tried to be classified according to the current Human Genome Variation Society (HGVS) nomenclature. In several cases, no American College of Medical Genetics and Genomics (ACMG) or International Agency for Research on Cancer (IARC) class was available.

Once the molecular genetic results are available, findings are discussed with the patient. In cases where a pathogenic or likely pathogenic variant is detected (classified as ACMG class 4 or 5), risk-reducing surgical options (e.g., mastectomy, adnexectomy) or enrollment in an intensified breast cancer surveillance (IBCS) are considered, depending on the specific gene mutation. If the variant is of uncertain significance (ACMG class 3), patients are advised to return for re-evaluation of the variant after three years. No immediate therapeutic or diagnostic measures are taken in such cases. A negative (non-pathogenic) result has no clinical implications. It should be emphasized that certain patient groups who meet the criteria for genetic testing may remain eligible for additional interventions even if their test results are negative or of uncertain significance. For instance, in Germany, breast cancer patients diagnosed before the age of 45 may participate in the IBCS program until they reach the age of 50, irrespective of genetic findings.

### Statistical analyses

Patient data from the period between January 1, 2018, and December 31, 2023, at University Hospital Erlangen were collected retrospectively. Medical histories and results of human genetic testing were documented in an electronic case report form (eCRF) using Microsoft Access 365 (Microsoft, Redmond, WA, USA). Descriptive analyses and statistical t-tests were performed using IBM SPSS Statistics, Version 31 (IBM Corp., Armonk, NY, USA).

## Results

### General patient characteristics

**Fig. 2:**
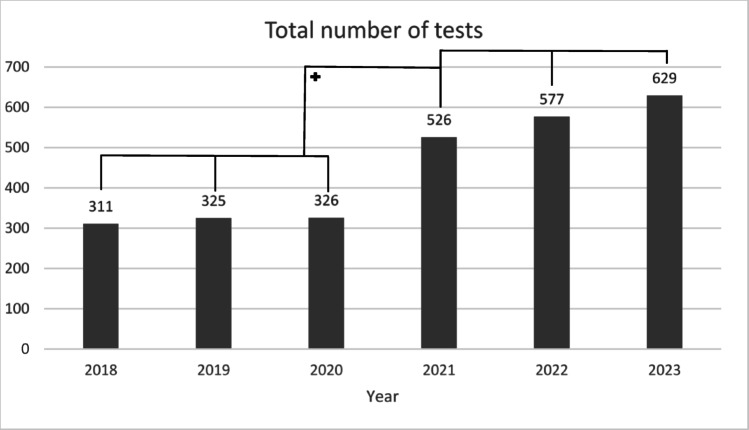
Number of genetic tests conducted at the HBOC center at the University Hospital Erlangen from 2018 to 2023. + = significant increase in the number of genetic testings is evident following certification in 2021 (*p* < 0.01)

At the center for HBOC at the University Hospital Erlangen, a total of 2694 clinical genetic tests were performed between 2018 and 2023. Prior to official certification (2018–2020), 962 genetic tests were conducted. Following certification (2021–2023), the number of tests increased to 1732, representing a significant 180.0% increase (*p *< 0,01) (Fig. 2.).

Between 2018 and 2020, predictive testing was carried out in 268 women, while diagnostic testing was performed in 625 women (Table 1). During the same period, 54 men underwent predictive testing and 5 men received diagnostic testing. After certification, from 2021 to 2023, 372 women were tested predictively and 1240 women diagnostically; additionally, 76 men underwent predictive testing and 10 men diagnostic testing (Table 1).

**Table 1:** Comparison of patient characteristics: sex and age distribution between 2018–2020 and 2021–2023

	2018–2020			2021–2023		
	Unaffected individuals (n/%)	Affected individuals (n/%)	No information available (n/%)	Unaffected individuals (n/%)	Affected individuals (n/%)	No information available (n/%)
Total number of tests	322	630	10	448	1250	34
Gender						
Female	268 (83.2%)	625 (99.2%)	9 (90%)	372 (83%)	1240 (99.2%)	33 (97.1%)
Male	54 (16.8%)	5 (0.8%)	1 (10%)	76 (17%)	10 (0.8%)	1 (2.9%)
Age						
< 35 years	105 (32.6%)	46 (7.3%)	2 (20%)	142 (31.7%)	72 (5.8%)	3 (8.8%)
≥ 35 years, < 50 years	100 (31.1%)	185 (29.4%)	2 (20%)	147 (32.8%)	343 (27.4%)	2 (5.9%)
≥ 50 years, < 70 years	105 (32.6%)	322 (51.1%)	6 (60%)	143 (31.9%)	640 (51.2%)	24 (70.6%)
≥ 70 years	12 (3.7%)	77 (12.2%)	0 (0%)	16 (3.6%)	195 (15.6%)	5 (14.7%)
Mean age (median age) in years	43.6 (42)	53.9 (54)	47.9 (51.5)	44.0 (42)	55.4 (56)	59.7 (61)

At the Gynecologic Cancer Center of the Department of Obstetrics and Gynecology, University Hospital Erlangen, 137 patients presented with a first diagnosis of ovarian cancer between 2018 and 2020, and 149 patients between 2021 and 2023 (Table 1). At the Breast Cancer Center of the same department, 1577 patients were newly diagnosed with breast cancer between 2018 and 2020, and 1856 patients between 2021 and 2023 (Table 1).

The mean age of women undergoing predictive testing was 43.6 years between 2018 and 2020, and 44.0 years between 2021 and 2023. For affected female patients, the mean age at testing was 53.9 years during 2018–2020 and increased to 55.4 years during 2021–2023.

### Genetic testing in breast and ovarian cancer

A total of 551 patients with breast cancer or ductal carcinoma in situ (DCIS) underwent genetic testing between 2018 and 2020, compared to 1104 patients during the period from 2021 to 2023 (Table 2). Diagnostic germline testing was performed in 117 patients with ovarian, fallopian tube, or primary peritoneal carcinoma between 2018 and 2020, and in 159 patients following certification from 2021 onward. In addition, starting in 2021, four patients with serous tubal intraepithelial carcinoma (STIC) were tested; none had been tested in the earlier period (2018–2020). Four patients with borderline ovarian tumors received genetic testing before certification, whereas 38 such patients were tested from 2021 onward.

The number of breast cancer patients in the lowest UICC stage (stage I) who underwent genetic testing increased by 132.4% following certification (2018–2020: 188 patients; 2021–2023: 249 patients) (Table 2). In UICC stage II, the number of tests increased by 198.3%, and in UICC stage III by 366.7%.

**Table 2:** Number of patients with different cancer types undergoing genetic testing and their clinical data (tumor stage according to UICC/FIGO) in comparison between 2018–2020 and 2021–2023

	2018–2020	2021–2023
	Affected individuals	Affected individuals
Breast cancer	513	1010
Carcinoma in situ of the breast	38	94
Ovarian cancer	100	148
Fallopian tube carcinoma	7	5
STIC	0	4
Borderline ovarian tumor	4	38
Primary peritoneal carcinoma	10	6
Breast cancer/ carcinoma in situ of the breast		
UICC 0	27	58
UICC I	188	249
UICC II	176	349
UICC III	33	121
UICC IV	16	102
No information available	109	188
Malignancies of the ovary, fallopian tube, and primary peritoneum		
FIGO I	19	18
FIGO II	3	6
FIGO III	52	76
FIGO IV	11	33
No information available	29	26

**In cases of multiple tumor entities, the highest tumor stage is considered

A total of 63 patients with ovarian cancer FIGO stage III or higher received diagnostic germline testing between 2018 and 2020, compared to 109 patients between 2021 and 2023, representing an increase of 173.0%.

### Genetic findings

A total of 962 genetic tests were conducted between 2018 and 2020, and 1732 tests between 2021 and 2023. Variants of uncertain significance (VUS) were identified in 86 cases prior to certification (8.9%) and in 168 cases after certification (9.7%) (Table 3).

Pathogenic findings were detected in 138 patients (14.3%) during the period 2018–2020, and in 199 patients (11.5%) from 2021 to 2023 (Table 3).

**Table 3:** Results of germline testing conducted in 2018–2020 and 2021–2023, classified according to ACMG/IARC guidelines in unaffected and affected patients

	2018–2020			2021–2023		
	Unaffected individuals (n/%)	Affected individuals (n/%)	No information available (n%)	Unaffected individuals (n%)	Affected individuals (n%)	No information available (n/%)
No or 1 (likely) benign variant (ACMG or IARC class 1 or 2)	205 (63.6%)	394 (62.5%)	7 (70%)	274 (61.2%)	904 (72.3%)	22 (64.7%)
1 variant of uncertain significance (ACMG or IARC class 3)	16 (5.0%)	70 (11.1%)	0 (0%)	26 (5.8%)	139 (11.1%)	3 (8.8%)
1 (likely) pathogenic finding (ACMG or IARC class 4 or 5)	20 (6.2%)	117 (18.6%)	1 (10%)	28 (6.3%)	166 (13.3%)	5 (14.7%)
*BRCA1/2* mutation	12	76	0	12	95	4
Non-*BRCA1/2* mutation	8	41	1	16	71	1
1 genetic variant, no ACMG or IARC class available	78 (24.2%)	24 (3.8%)	2 (20%)	110 (24.6%)	10 (8.0%)	4 (11.8%)
*BRCA1/2* mutation	58	10	2	84	5	3
Non-*BRCA1/2* mutation	20	14	0	26	5	1
2 genetic variants	3 (1.0%)	25 (4.0%)	0 (0%)	10 (2.2%)	31 (2.4%)	0 (0%)

In 2018–2020, a pathogenic *BRCA1/2* mutation was confirmed in 88 patients (9.2%). An additional 70 patients were found to carry a *BRCA1/2* mutation, though these variants were not classified at the time of testing. Altogether, *BRCA1/2* mutations were detected in 158 patients, corresponding to 16.4% of all tested cases during that period (Table 3).

From 2021 to 2023, a pathogenic *BRCA1/2* mutation was identified in 111 patients (6.4%). Another 92 patients carried unclassified *BRCA1/2* mutations. In total, *BRCA1/2* mutations were observed in 203 cases, representing 11.7% of all tested patients.

### Clinical consequences

**Fig. 3:**
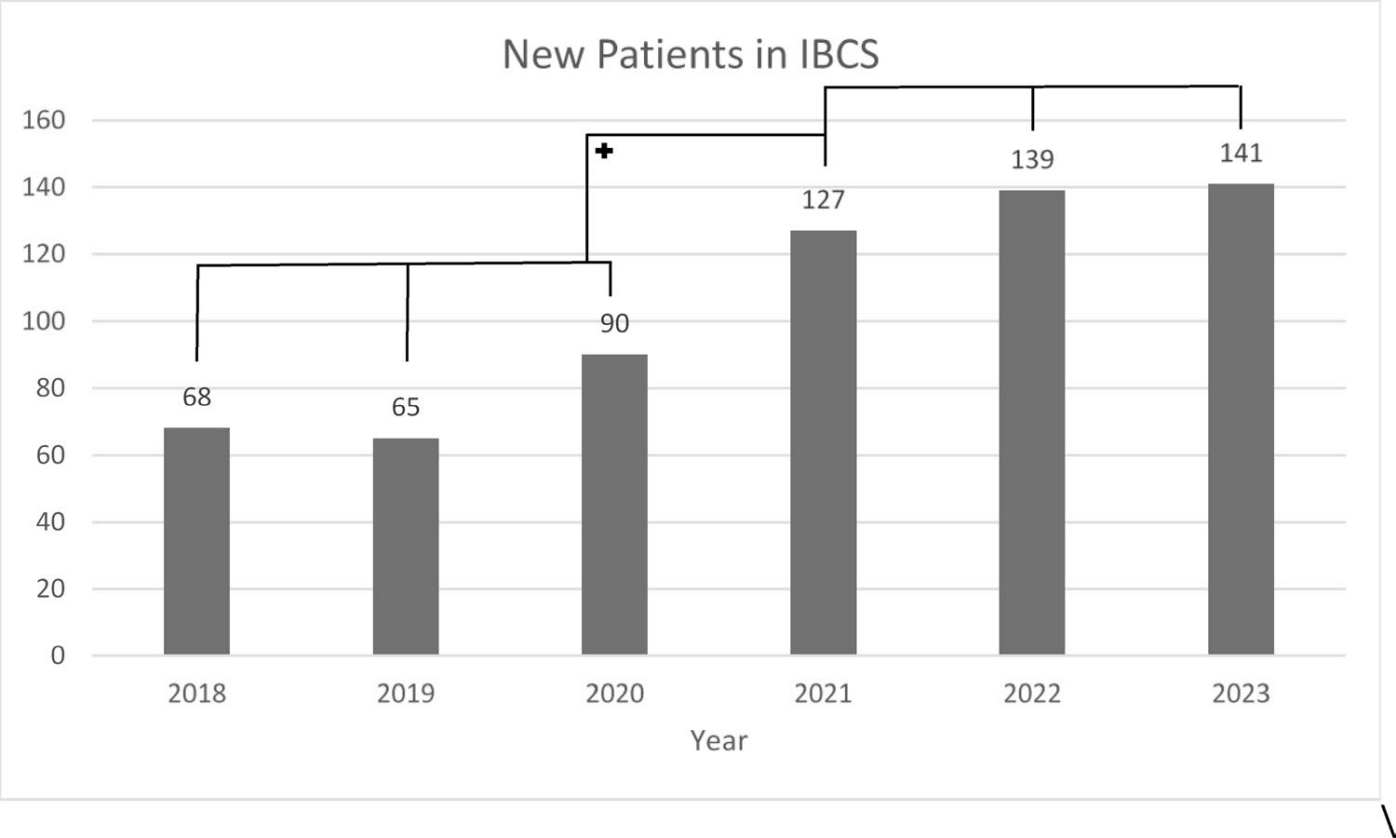
Number of new female patients enrolled in the IBCS program due to a familial high-risk situation, stratified by affected and unaffected status, from 2018 to 2023. + = significant increase in the number of genetic testing is evident following certification in 2021 (*p* < 0.02)

Between 2018 and 2020, 223 patients were enrolled in the IBCS program. Following certification, 407 patients were included, representing an increase of 182.5% (Fig. 3).

After certification, enrollment of affected patients into the IBCS program increased by 205.8% compared to the pre-certification period. Among unaffected individuals, the increase amounted to 141.3%.

Regarding to risk reducing surgery between 2018 and 2020, RRSO was recommended to 127 patients, and RRBM was advised for 111 patients. From 2021 to 2023, recommendations for RRSO were given to 159 patients, while 146 patients were advised to undergo RRBM (Table 4).

**Table 4:** Enrollment numbers of affected and unaffected patients in the IBCS program and the number of recommendations for risk-reducing surgeries (RRBM RRSO) compared between 2018–2020 and 2021–2023

	2018–2020			2021–2023		
	Unaffected individuals (n%)	Affected individuals (n/%)	No information available (n/%)	Unaffected individuals (n/%)	Affected individuals (n/%)	No information available (n/%)
Intensified breast cancer surveillance						
Total Numbers	75	146	2	106	299	2
1 (likely) pathogenic *BRCA1/2* mutation (ACMG or IARC class 4 or 5)	8 (10.7%)	53 (36.3%)	0 (0%)	11 (10.4%)	62 (20.7%)	1 (50%)
1 (likely) pathogenic non-*BRCA1/2* mutation (ACMG or IARC class 4 or 5)	5 (6.7%)	17 (11.6%)	0 (0%)	13 (12.3%)	50 (16.7%)	0 (0%)
No genetic variant or 1 non-pathogenic variant (ACMG or IARC class 1–3)	5 (6.7%)	53 (36.3%)	1 (50%)	12 (11.3%)	168 (56.2%)	0 (0%)
1 genetic variant, no ACMG or IARC class available	53 (70.7%)	10 (6.9%)	1 (50%)	63 (59.4%)	6 (2.0%)	1 (50%)
2 genetic variants	4 (5.3%)	13 (8.9%)	0 (0%)	7 (6.6%)	13 (43.4%)	0 (0%)
RRSO						
Total Numbers	55	71	1	75	83	1
1 (likely) pathogenic *BRCA1/2* mutation (ACMG or IARC class 4 or 5)	8 (14.5%)	52 (73.2%)	0 (0%)	11 (14.7%)	57 (68.7%)	1 (100%)
1 (likely) pathogenic non-*BRCA1/2* mutation (ACMG or IARC class 4 or 5)	3 (5.5%)	6 (8.5%)	0 (0%)	2 (2.7%)	13 (15.6%)	0 (0%)
No genetic variant or 1 non-pathogenic variant (ACMG or IARC class 1–3)	1 (1.8%)	0 (0%)	0 (0%)	0 (0%)	0 (0%)	0 (0%)
1 genetic variant, no ACMG or IARC class available	40 (72.7%)	6 (8.5%)	1 (100%)	57 (76%)	6 (72.3%)	0 (0%)
2 genetic variants	3 (5.5%)	7 (9.8%)	0 (0%)	5 (6.6%)	8 (66.4%)	0 (0%)
RRBM						
Total Numbers	50	60	1	69	77	0
1 (likely) pathogenic *BRCA1/2* mutation (ACMG or IARC class 4 or 5)	8 (16%)	45 (75%)	0 (0%)	11 (15.9%)	59 (76.6%)	0
1 (likely) pathogenic non-*BRCA1/2* mutation (ACMG or IARC class 4 or 5)	0 (0%)	1 (1.7%)	0 (0%)	2 (2.9%)	7 (9.1%)	0
No genetic variant or 1 non-pathogenic variant (ACMG or IARC class 1–3)	0 (0%)	0 (0%)	0 (0%)	0 (0%)	0 (0%)	0
1 genetic variant, no ACMG or IARC class available	39 (78%)	6 (10%)	1 (100%)	53 (76.8%)	4 (5.2%)	0
2 genetic variants	3 (6%)	8 (13.3%)	0 (0%)	3 (4.4%)	7 (9.1%)	0

## Discussion

Through the certification of oncological and non-oncological centers, patient care is intended to be safeguarded and continuously improved based on high-quality indicators (Beckmann, et al. 2007). The German Cancer Society has played a pioneering role in this field for many years. Designation as a certified center for hereditary breast and ovarian cancer (HBOC) has markedly increased the number of patients undergoing genetic counseling and testing at University Hospital Erlangen. Notably, more patients with advanced breast or ovarian cancer were offered the opportunity to explore additional therapeutic options through molecular diagnostics. Furthermore, unaffected individuals gained access to preventive strategies, including risk-reducing interventions and enrollment in intensified early detection programs, thereby lowering their cancer risk.

Adequate oncological care requires interdisciplinary and interprofessional collaboration across internal and external networks (Wesselmann et al. 2014b). The certification of oncological institutions ensures high-quality treatment based on evidence-based clinical guidelines and corresponding performance indicators (Wesselmann et al. 2014b; Deckert et al. 2021). Patients treated at certified centers demonstrate improved overall and disease-free survival (Schmitt et al. 2023). Numerous studies support these findings: patients with breast cancer or colorectal carcinoma treated at certified centers showed significantly improved prognosis due to adherence to standardized, quality-assured treatment protocols (Beckmann et al. 2011; Bierbaum et al. 2024; Jacob et al. 2021; Kreienberg et al. 2018; Kowalski et al. 2012). Similarly, patients with head and neck tumors or pancreatic cancer achieved better outcomes in certified hospitals (Modabber et al. 2021; Roessler et al. 2022).

However, patient volume alone does not appear to be the sole determinant of treatment quality. Ortmann et al. evaluated 193 certified centers for gynecologic malignancies and reported that only low-volume centers (fewer than 15 ovarian cancer cases annually) were associated with poorer patient outcomes (Ortmann et al. 2025). Among centers treating more than 15 ovarian cancer cases per year, no significant differences in survival outcomes were observed, irrespective of case volume (Ortmann et al. 2025). There appears to be a ceiling effect regarding case volume. A certain amount of patients are needed to ensure a high quality treatment, above a certain threshold ever more patients do not attribute to improved outcomes.

To guarantee optimal care for HBOC patients, a structured, multi-institutional model of care was initiated in Germany in 1996 (Schmutzler, et al. 2005). Over time, this initiative expanded to more than 12 specialized centers and, in 2005, was integrated into standard healthcare services with reimbursement by statutory health insurance providers (Schmutzler, et al. 2005). A key strength of this model is its foundation in knowledge-generating healthcare, allowing diagnostic and preventive strategies to be continuously adapted in line with emerging evidence (Kast, et al. 2020). Regular evaluations facilitate the incorporation of novel findings into clinical recommendations. Importantly, uniform quality standards are maintained across all participating sites through standardized frameworks and close inter-center collaboration, regardless of patient volume (Kast, et al. 2020). International partnerships further promote cross-border cooperation in research and clinical management (Kast, et al. 2020). The GCHBOC is a member of the BCAC (Breast Cancer Association Consortium) and CIMBA (Consortium of Investigators of Modifiers of *BRCA1/2*) networks, international consortia for hereditary breast cancer. As by far the most productive consortium, the GCHBOC plays a leading role in implementing care standards and knowledge-generating research. Structured processes in patient care and objective monitoring, such as the annual certification audits conducted in Germany, are not yet established internationally. Initiatives like the CIMBA network aim to promote high-quality patient care on a global scale.

Expansion of the HBOC consortium was accompanied by the development of a regional network of certified breast and gynecologic cancer centers, ensuring high-quality, guideline-based care in close proximity to patients’ homes and thereby improving accessibility and adherence (Kowalski et al. 2017).

Following its designation as a certified HBOC center, the University Hospital Erlangen expanded its network of collaborating gynecology clinics in the surrounding region, which contributed to increased patient referrals. More decisive, however, were the approvals of targeted therapies for early-stage breast and ovarian cancer (Ray-Coquard et al. 2019; Tutt et al. 2021). Following the introduction of PARP (Poly-(ADP-ribose)-Polymerase) inhibitors for the treatment of metastatic or locally advanced HER2-negative breast cancer associated with pathogenic *BRCA1/2* mutations, germline testing also gained therapeutic relevance. The numbers were rising. On parallel, testing criteria defined by the German Cancer Society have undergone continuous evaluation and expansion. With the steadily expanding inclusion criteria, the number of patients who do not meet the classic HBOC criteria based on their original family history is increasing, for example in the therapeutic setting. This, in turn, can lead to a decrease in the relative proportion of pathogenic mutations in the overall cohort. Indeed, the relative proportion of pathogenic mutations decreased from 14.3 to 11.5% between 2021 and 2023, despite an increase in the number of tests performed.

Patients generally place high value on treatment quality, accessibility, and certification (Lux et al. 2011). Individuals from rural regions appear particularly willing to accept longer travel distances to access specialized care (Schirrmacher et al. 2022). Systematic strengthening of structural and outcome quality through certification processes has further enhanced trust in certified institutions. Annual evaluations ensure the maintenance of high medical standards, a factor of particular importance for oncology patients. These aspects collectively help explain the substantial increase in patient numbers observed in both genetic testing and intensified early detection programs.

Nevertheless, the rise in patient numbers following official certification does not necessarily translate into improved quality but rather into increased workload and procedural demands. Intensified early detection strategies include annual breast MRI (Magnet Resonance Imaging), supplemented by mammography and ultrasound when indicated (Bick 2021; Bick et al. 2019; Madorsky-Feldman et al. 2016). Breast MRI is resource-intensive, requiring significantly greater personnel and time compared to mammography. Higher patient volumes may also entail additional organizational efforts, and infrastructural measures, such as the acquisition of new radiological equipment, are often implemented with delay. Prolonged waiting times may, in turn, reduce patient satisfaction. Thus, a potential decline in quality cannot be entirely excluded.

From a health-economic perspective in particular, diagnostic and therapeutic interventions should be indicated in a resource-efficient manner, weighing costs against benefits. For example, careful consideration of potential participation in the IBCS should be limited to specific risk constellations. For high-risk patients with *BRCA1/2* mutations, the number needed to treat is 1:100 (Zelst et al. 2017). For patients with moderate or low risk, or those with unremarkable genetic findings but a relevant family history, the number needed to treat is higher. Further analyses from large-scale registry studies are needed to provide evidence-based recommendations, or to withhold interventions, for these patient groups.

Rising numbers in patients (genetic testing, surveillance program) observed in an HBOC center following successful initial certification are expected to occur in other German HBOC centers as well. The first certification in Erlangen was granted in 2021, making it one of the pioneers in Germay. This objective assessment of structural, process, and outcome quality is already been transfered to other clinical institutions. Following successful certification, HBOC centers should anticipate an increase in patient numbers, along with associated new personnel and organizational challenges.

### Strengths and limitations

Within the context of health policy regulations and ongoing hospital reforms, the certification of healthcare institutions is essential. It serves to ensure patient care based on high-quality indicators. This is made possible through objectively assessed and evidence-based criteria. For the first time, to our knowledge, the present study has investigated the impact of certification processes in HBOC centers. The analyses was also focused on differences in patient characteristics and tumor-specific features. However, data collection was limited to a single center. Consequently, generalization to the overall German patient population is only possible to a limited extent. Further studies, particularly with regard to tumor-specific characteristics, are therefore required to provide a more comprehensive representation.

## Conclusion

Certification helps ensure that the quality of patient care and treatment is maintained at a consistently high, evidence-based level. High-quality medical care is particularly essential in times of centralization processes driven by health-economic cost pressures, in order to emphasize to patients the relevance of receiving treatment at specialized centers, even in the face of potentially longer travel distances. Quantitatively, certification is reflected in an increased number of patient referrals. Furthermore, additional services, particularly preventive measures for HBOC, are more frequently utilized at these centers. In times of hospital reforms and structural changes within the healthcare sector, certification of medical institutions can provide reassurance for both treating physicians and patients. Further studies are warranted to emphasize the necessity and impact of hospital certification.

## Supplementary Information

Below is the link to the electronic supplementary material.

Supplementary file1 (DOCX 17 kb)

## Data Availability

Data that support the findings of this study are available from the corresponding author upon reasonable request.

## Abbreviations

ACMG: American college of medical genetics and genomics
BCAC: Breast cancer association consortium
BRCA: Breast cancer gene
CIMBA: Consortium of investigators of modifiers of BRCA1/2
DCIS: Ductal carcinoma in situ
eCRF: Electronic case report forms
FIGO: Fédération internationale de gynécologie et d’obstétrique
GCHBOC: German consortium for familial breast and ovarian cancer
HBOC: Hereditary breast and ovarian cancer
HER: Human epidermal growth factor receptor
HGVS: Human genome variation society
IARC: International agency for research on cancer
IBCS: Intensified breast cancer surveillance
MRI: Magnetic resonance imaging
NGS: Next generation sequencing
OPD: Outpatient department
PARP: Poly-(ADP-ribose)-polymerase
RRBM: Risk-reducing bilateral mastectomy
RRSO: Risk-reducing salpingo-oophorectomy
STIC: Serous tubal intraepithelial carcinoma
UICC: Union internationale contre le cancer
WiZen: Wirksamkeit der versorgung in onkologischen zentren
