# Supplementary material for: Quality of care before and after initial certification at a German certified hereditary breast and ovarian cancer center
Source: J Cancer Res Clin Oncol. 2025 Dec 17;152(1):13. doi: 10.1007/s00432-025-06388-3 (PMC12711623; doi:10.1007/s00432-025-06388-3)
Supplement: Supplementary file 1 — Supplementary file1 (DOCX 17 kb) [file 432_2025_6388_MOESM1_ESM.docx]

**Supplementary Information**

| Quality indicators HBOC |
| --- |
| Basic data (Affected, Non-affected persons, Cooperation partners) |
| Detection rate in IBCS |
| Histologically confirmed breast cancer and/or DCIS following BI-RADS 4/5 findings in IBCS |
| Stage distribution of diagnosed breast carcinoma/DCIS in IBCS |
| Proportion of positive findings following core needle biopsy in the IBCS |
| Proportion of mutation detection, class 4/5 |
| Number of studies conducted |
| Number of study enrollments in HerediCaRe |

SI 1. Quality indicators HBOC for Onkozert certification process

| Breast and ovarian cancer genes | 2018-2020 | 2021-2023 |
| --- | --- | --- |
| ATM | 16 | 27 |
| BARD1 | 2 | 4 |
| BRCA1 | 90 | 116 |
| BRCA2 | 72 | 106 |
| BRIP1 | 7 | 10 |
| CDH1 |  | 2 |
| CHEK2 | 31 | 43 |
| EPCAM |  | 1 |
| MLH1 | 1 | 2 |
| MRE11A | 1 |  |
| MSH2 | 1 | 12 |
| MSH6 | 1 | 7 |
| MUTYH | 1 |  |
| NBN | 3 | 2 |
| NF1 |  | 1 |
| NTHL1 |  | 1 |
| PALB2 | 15 | 25 |
| PMS2 | 6 |  |
| PTEN | 1 | 2 |
| RAD51C | 1 | 3 |
| RAD51D | 5 | 2 |
| TP53 | 2 | 2 |
| VHL | 1 |  |

SI 2. Breast and ovarian cancer genetic variants of lWIST Exome 2.0 Enrichment Technologie (lWIST Bioscience) between 2018-2023.
